# A Nursing Faculty Intervention for Teaching Primary Palliative Care in Baccalaureate Nursing Competency-Based Education

**DOI:** 10.1089/pmr.2024.0079

**Published:** 2025-02-10

**Authors:** Olga Ehrlich, Julie A. Kruse, Alyssa Lackowski, Toni L. Glover

**Affiliations:** Oakland University School of Nursing, Rochester, Michigan, USA.

**Keywords:** competency-based education, faculty education intervention, nursing education, primary palliative care

## Abstract

**Background:**

The American Association of Colleges of Nursing introduced hospice/palliative/supportive care as a focus area for baccalaureate nursing and identified pre-licensure competency-based education as essential for a safe and effective entry-to-practice nurse workforce. Yet many nursing educators lack knowledge and skills to teach competency-based primary palliative care (PPC).

**Method:**

We piloted an educational intervention for baccalaureate nursing faculty in developing a competency-based PPC learning activity. The intervention consisted of workshops during which pre- and posttest data were collected. Intervention materials included an open-access PPC faculty resource *Guide* and a *Learning Activity Template* developed by the first author.

**Results:**

Paired samples *t* tests demonstrated significant knowledge gains and changes in attitudes post-intervention in 17 of 20 areas assessed including “I am confident in my ability to teach nursing students about end-of-life care.” There was a statistically significant increase in the total palliative care score (all 20 areas assessed) from pretest (*M* = 70.25, standard deviation [*SD*] = 9.54) to posttest (*M* = 80.40, *SD* = 10.10), *t* (39) = 9.18, *p* < 0.001 [two-tailed]). The Cohen’s *d* statistic (1.45) indicated a large effect size. Additionally, over half of the participants created unique learning activities (*n* = 22) for teaching competency-based PPC.

**Conclusion:**

This intervention addresses a faculty need in the context of new accreditation expectations. The open-access intervention materials shared in this article can be used to create course learning activities aligned with nursing needs for competency-based PPC. Future research should focus on effective faculty teaching interventions and reliable and valid assessment tools for faculty needs and student competency.

## Introduction

In 2021, the American Association of the Colleges of Nursing (AACN) released a revised *Essentials* document to guide quality and provide standards in nursing education programs. Major changes included a competency-based education (CBE) focus and a new requirement that baccalaureate nursing students demonstrate competency in hospice/palliative/supportive care.^1^ To meet the new *Essentials* standard of integrating hospice/palliative/supportive care into curriculum, faculty require access to evidence-based resources and knowledge of how to teach primary palliative care (PPC) according to national standards and CBE principles.^1^

Palliative care is the provision of supportive interdisciplinary services to address and ameliorate discomforting symptoms for persons with serious illness of any age and in any setting. The intention is to promote physical, psychosocial, emotional, and spiritual well-being and interventions that are aligned with patient and family values. Palliative care is considered specialty health care and can be provided concurrently with curative interventions. Hospice care is a part of palliative care and focuses on symptom management and quality of life for persons with a prognosis of six months or less, and who are not pursuing curative treatments, or there are no further curative treatment options.^2,3^

PPC nursing applies holistic assessment and intervention to relieve suffering and improve quality of life across the lifespan, illness trajectory, and setting and can be delivered by nonspecialty nurses.^2,3^ The World Health Organization declared that all health care systems are ethically responsible for providing palliative care.^4^ However, palliative care is not equitably available across clinical settings or communities. For example, rural communities with small hospitals are less likely to have palliative care than in urban areas.^5,6^ Good communication plays a key role in palliative care provision. Palliative communication competency includes asking patients questions about what matters most to them, what they would prioritize if they become sicker, or assisting in identifying a surrogate decision-maker and choices for end-of-life care. Without palliative care, patients may experience preventable or unwanted hospitalizations for symptom management or illness exacerbations.^6^ When nurses have education and experience in using palliative communication, clarification, and congruence of patient values and treatment can reduce emotional burden for patients, families, and nurses.^7–9^ This is notable because nurses across practice settings have been challenged to meet PPC needs for patients with serious illness.^2^ The American Academy of Nursing Expert Panels made recommendations for improving palliative care access internationally, including expanding palliative care nursing education and nurses educating interdisciplinary colleagues, the public, and policy makers about positive patient and family outcomes of palliative care.^10^ However, nursing education programs have been slow to integrate PPC. The End-of-Life Nursing Consortium (ELNEC) reports that ∼135 of 996 baccalaureate nursing programs in the United States offered standardized PPC education from 2021 to 2023.^11,12^ Challenges to integration of PPC content in nursing education include a general PPC faculty knowledge gap and a paucity of resources for educating faculty to teach and incorporate PPC content. Thus, prelicensure faculty education and training interventions are needed to meet this call for PPC nursing.

The foremost resource for teaching pre-licensure nursing students PPC has been the ELNEC Undergraduate/New Graduate online curriculum.^13^ These evidence-based textual modules, videos, quizzes, and tests were developed and revised by palliative/hospice care nurse experts in alignment with PPC competencies outlined in the *Competencies and Recommendations for Educating nursing Students* (*CARES*).^14–16^ More than 150 nursing programs in the United States have used the ELNEC modules which are free for nursing faculty and charge students a modest fee.^11^ The ELNEC modules provide a strong introduction to evidence-based PPC, focusing on knowledge, one element of CBE.^11^ However, an important gap between ELNEC undergraduate/new graduate and student achievement of PPC competencies is the lack of leveled CBE learning activities, critical to the development of student competency over time. Competency is evident when nursing students embody what nurses do by synthesizing knowledge, skills, and attitudes, rather than identifying what it is that nurses know.^17–19^

Since existing PPC materials are largely knowledge versus competency based and knowing that PPC competencies need to be threaded throughout the curriculum, nursing educators without PPC expertise require guidance in developing scaffolded CBE PPC course materials. To meet this need, the first author developed an educational intervention for faculty about best-practice resources in PPC and developing a PPC CBE course learning activity. A guidebook, *Integrating the AACN Hospice/Palliative/Supportive Care Sphere in Undergraduate Nursing Education: A Practical Guide for Faculty*, hereafter referred to as the *Guide*, was used during workshops to orient faculty to PPC resources that can be integrated into courses.^20^ To our knowledge, this was the first faculty-focused intervention used to build PPC teaching and CBE capacity in baccalaureate nursing education using a standardized faculty education module and resource guide for the development of a learning activity.^21^ This article reports the results of piloting the faculty intervention which also included education about CBE, the *Essentials*,^1^ and palliative care definitions.

## Methods

### Design

This study was an education intervention pilot program with a descriptive pretest–posttest design. Data were collected from January to May of 2024 at one face-to-face workshop and two online workshops.

### Participants

Faculty who taught undergraduate nursing courses were eligible. Since the intervention content was presented in English, the only exclusion criteria to participate was not being able to communicate in English. Forty participants consented and were enrolled in the study.

### Setting

The intervention included in-person workshops at the authors’ school and an online option for remote attendees. Workshops were publicized via email invitations, at faculty assembly meetings, via national nursing organization listservs (AACN and HPNA), and by word-of-mouth through the first author’s professional networks, such as faculty colleagues at other universities. Study activities were conducted during the intervention workshops. For online participants, virtual breakout rooms were created to facilitate interaction during activities. Pre- and posttest surveys were administered online using Qualtrics (https://www.qualitrics.com).

### Ethical considerations

The authors’ institutional review board approved this study (OUIRB-FY2023-104). Consent-to-participate was completed via an online form prior to completing the pre- and posttests. For privacy and matching of pretest and posttest survey responses, each participant created and used a unique identifier.

### Procedure

Faculty were invited to participate in the educational workshop using the methods described previously; however, recruitment to complete the pre- and posttest surveys occurred during the intervention workshop welcome. Thus, study participants comprised a convenience sample of self-selected faculty at each of the three workshops. Participants completed the pre- and posttest surveys prior to the start of the workshop and at the conclusion of the workshop respectively. In-person workshop participants received continuing education credits as compensation, whereas virtual participants could opt into a drawing to win one of four palliative care textbooks.

The study intervention consisted of a one-time, three-hour-long interactive workshop to educate faculty about evidence-based, no-cost educational resources in PPC and to develop a PPC CBE course learning activity. Workshop content included a review of the *Essentials* and competencies/sub-competencies related to PPC,^1^ introduction to the *Guide*,^20^ and a *Learning Activity Template*.^21^ The primary goal of the workshop and study outcome was to aid faculty in developing a competency-based learning activity for their course which met *Essentials* competencies/sub-competencies.^1^ The *Learning Activity Template* was the documentation tool used to determine whether this outcome was achieved.^21^

An internal Oberhauser Award from the Oakland University School of Nursing paid for workshop materials, which remain available in perpetuity as open-source digital documents and videos.^20–22^ The workshop content was developed by the first author, a board-certified hospice and palliative care nurse, holding a teaching certificate in nursing education and with experience teaching interdisciplinary clinical palliative care. The first author presented the workshops three times, using a standardized script. The first workshop was in-person for faculty at the authors’ institution. Two co-authors were participants in the faculty workshop (J.A.K. and T.L.G.). The second and third workshops were presented via a synchronous online platform. The workshop format was a live slide presentation with the instructor on camera. The agenda was as follows: introduction and invitation to participate in study; survey completion for study participants; review of objectives; participation question: “What do you think of when you hear palliative care’?” with chat responses; formal definitions of palliative care and hospice mini lecture; overview of AACN *Essentials*^1^ mini-lecture; chat-based poll “What do you know about ELNEC CARES^14,15^ competencies?”; example of integrated CARES^14,15^ and *Essentials*^1^ domain/competency/subcompetency; breakout room exercise one (10 minutes to identify a domain/competency/subcompetency for use in learning activity); breakout room exercise two (20 minutes to look over the *Guide*^20^ and select a topical area and resource to use in learning activity development); CBE mini-lecture; breakout room exercise three (60 minutes to work independently developing a learning activity using the *Learning Activity Template*^21^); large group discussion participant sharing of content developed in breakout sessions; posttest survey for study participants; workshop conclusion. The workshop remains available for use as a 3-hour self-paced online learning unit.^22^

The *Guide* is an open-source PPC guidebook created to introduce baccalaureate nursing faculty to a wide range of learning activities and evidence-based sources.^20^ All *Guide* resources are cost-free, enabling faculty to develop or incorporate learning activities without adding to student out-of-pocket expenses.^20^ A *Learning Activity Template* was designed to walk participants through selecting a domain, competency, and sub-competency from the *Essentials*;^1^ selecting a PPC topical area and resource from the *Guide*;^20^ and developing a course learning activity.^21^ Study participants were taught how to use the *Learning Activity Template* in three phases, each of which scaffolded on the previous phase content (e.g., first selecting an *Essentials* domain,^1^ then selecting a content topic and *Guide* resource, followed by developing a unique learning activity).^20,21^

### Instruments

The first author developed surveys based on the objectives of the workshop to assess faculty’s knowledge and attitudes/beliefs about PPC and perceived ability to teach PPC nursing. The pretest and posttest surveys included 20 identical items. To ensure face and content validity of the tools, the initial pre- and posttest surveys were revised based on input from three baccalaureate nursing faculty who maintain hospice and palliative nurse certification. Reliability was tested prior to the implementation of the survey with 22 baccalaureate nursing faculty at the authors’ institution, and the surveys were determined to have good reliability (Cronbach’s alpha 0.908) (O Ehrlich & K Moxley, 2023, unpublished data). For this study, the pretest Cronbach’s alpha = 0.821 and the posttest Cronbach’s alpha = 0.919.

An additional three questions on the pretest survey elicited specialty training, previously taught PPC course content, and PPC teaching needs. Ten additional items on the posttest survey related to the usefulness of the workshop and *Learning Activity Template* are listed in Table 1.^21^ Additionally, demographic data were obtained including participant educational background, institution name and location, and previous formal or informal education, training, or life experiences with palliative care.

**Table 1.** Pretest and Posttest Survey Items for Helpfulness and Learning Activity Development

Question category
Helpfulness
Item (Likert scale responses “0-not at all” to “5-completely”)
By participating in this workshop, I was able to develop a primary palliative care CBE learning activity for a course I teach or plan to teach.
By participating in this workshop, I have increased my knowledge about one component of primary palliative care education
I could use the Guidebook to develop primary palliative care CBE learning activity in the future.
Learning Activity Template
Item (Free text response)
Please copy and paste your response from the Learning Activity Template, question 3, Essentials Domain selected:
Please copy and paste your response from the Learning Activity Template, question 4, Essentials Competency selected:
Please copy and paste your response from the Learning Activity Template, question 5, Essentials sub-competency selected:
Please copy and paste your response from the Learning Activity Template, question 7, Learning Activity topic area (e.g., pain management, advance care planning, and heart failure).
Please copy and paste your response from the Learning Activity Template, question 8, Learning Activity proposed name (e.g., use of opioids for dyspnea relief in end-stage COPD):
Please copy and paste your response from the Learning Activity Template, question 9, Guidebook resource being used to support the learning activity: a) Name of resource: b) Page resource is located on:
Please provide a brief description of the learning activity you created during the workshop.

CBE, competency-based education.

An *a priori* power analysis was conducted using an online statistical calculator, Statulator, by Dhand and Khatkar.^23^ The sample size needed to achieve 80% power for detecting a medium effect and a significance level of α = 0.05 was *n* = 34. Therefore, our sample size of *n* = 40 is adequate to test our hypothesis.

### Analysis

Data were analyzed using IBM SPSS software (version 29). Only 0.49% of pretest and 1.34% of posttest items were missing from the dataset because they were unanswered. A nonsignificant Little’s MCAR test, χ2(76) = 90.90, *p* = 0.117 (pretest) and *χ*2(66) = 46.05, *p* = 0.971 (posttest) revealed that the data were missing completely at random.^24^

When <5% of data are missing and if these data are missing completely at random, then imputation using variable means produces unbiased parameter estimates and is considered acceptable.^25^ Mean imputation is also supported by Peng et al. who assert this method can be used when no >10%–20% of data are missing.^26^ Tabachnick and Fidell additionally highlight that almost any procedure for handling missing data (if <5%) will produce similar results if the data are missing completely at random.^25^ In this study, missing data were imputed using individual variable means. Variables were analyzed using counts and percentages. Paired samples *t* tests were used to compare differences in pre- and posttest scores. A priori content analysis was used to categorize and describe the data obtained from the open-ended survey questions.

## Results

There were 66 workshop participants, representing 102 nursing institutions from 36 (72%) U.S. states. Workshop participants reported a range of terminal degrees from baccalaureate to doctorate degrees, with 44 (66%) having earned a doctorate, 21 (38%) a master’s degree, and 1 (2%) a baccalaureate degree. Specialty certification in palliative care was reported by 5 (8%) participants. Additionally, 24 (36%) attendees reported either specialty training, nursing experience, or personal experience in palliative care or pain management. No training or palliative care experience was reported by nine participants (13%) and 14 (21%) did not answer the question.

The paired-samples *t test* revealed 17 statistically significant pairs of results with 7 pairs of results with a Cohen’s *d* effect size >0.5 (but <0.8) indicating a moderate effect and 3 pairs with an effect size >1.0 indicating a large effect (see Table 2). Confidence in ability to teach nursing students about end-of-life care and in ability to define palliative care nursing both had large effect sizes as well as “I am familiar with learning materials and resources that an instructor of nursing can incorporate into coursework to discuss various palliative care nursing topics.” Additionally, the total scale score for the pretest was compared to the total scale score for the posttest with statistically significant results with the Cohen’s *d* = 1.43 indicating that the intervention had a large effect (Table 2).

**Table 2.** Paired Samples *t*-Test Comparison of Pre- and Post- Survey Scores for Faculty Who Completed Palliative Care Trainings (*n* = 40)

	Pre-test		Post-test				
Parameter	M	SD	M	SD	*t*	*p*	Cohen’s *d*
I understand the differences between palliative care and hospice.	3.93	1.02	4.53	0.64	−3.59	<.001	.567
I am confident in my ability to teach nursing students about end-of-life care.	3.15	1.31	4.13	0.79	−6.33	<.001	1.001
I am confident in my ability to define palliative care nursing.	3.58	1.01	4.55	0.60	−6.92	<.001	1.094
I understand and could explain advance care planning to a friend or family member.	3.68	1.21	4.08	1.10	−3.40	<.001	.537
I believe that all registered nurses are capable of learning how to deliver primary (nonspecialist) palliative care interventions.	4.33	1.00	4.63	0.63	−2.02	.050	.319
I believe that all palliative care nursing (both primary and specialty) should only be delivered by nurses who have specialist palliative care training.	1.48	1.43	1.43	1.84	0.19	.847	.031
I am ready to incorporate the concept of primary palliative care nursing as it applies to objectives and content in one of my nursing classes.	4.00	1.09	4.38	0.63	−2.42	.020	.383
I believe that primary palliative care nursing can be incorporated into a variety of nursing classes.	4.43	0.93	4.85	0.36	−2.81	.008	.444
I am familiar with learning materials and resources that an instructor of nursing can incorporate into coursework to discuss various palliative care nursing topics.	2.25	1.26	4.35	0.58	−9.42	<.001	1.489
I believe that palliative and end-of-life care are specialties that detract from more important nursing content.	0.33	0.76	0.80	1.64	−1.84	.073	.291
I have the knowledge to teach pain assessment to nursing students.	4.03	0.73	4.23	0.83	−1.84	.073	.291
I have the knowledge to teach individualized pain management interventions to nursing students.	3.80	0.82	4.18	0.81	−4.39	<.001	.694
I have the knowledge to teach symptom assessment in a variety of contexts to nursing students.	3.78	0.77	4.13	0.94	−2.56	.014	.405
I have the knowledge to teach dyspnea assessment and nursing interventions to nursing students.	3.53	1.04	4.08	0.94	−4.27	<.001	.675
I have the knowledge to teach patient-centered empathetic nursing communication skills to students in the context of one of my courses.	3.90	0.78	4.33	0.69	−3.98	<.001	.630
I am comfortable talking about dying when teaching nursing care	4.23	0.86	4.43	0.78	−2.08	.044	.329
I am prepared to teach care of the dying hospitalized patient in a nursing course.	3.50	1.11	4.05	0.93	−4.44	<.001	.703
I am comfortable talking about grief, bereaving processes, and loss.	3.88	0.99	4.18	0.81	−2.62	.012	.415
I can name three serious illnesses (chronic life-limiting conditions) for which patients could benefit from palliative care.	4.65	0.62	4.85	0.36	−2.73	.010	.431
I have the knowledge to incorporate cultural sensitivity into curricular content.	3.85	0.62	4.28	0.62	−3.60	<.001	.569
Palliative Care Total Score	70.25	9.54	80.40	10.10	−9.18	<.001	1.45

SD, standard deviation.

### Intervention completion

Intervention completion was determined by the rate of participants who reported completing the *Learning Activity Template*.^21^ All 40 participants responded to a posttest survey item, “By participating in this workshop, I was able to develop a PPC CBE learning activity.” Totally, 22 participants (55%) selected “5-completely” for completion, with 13 (33%) responding, “4,” four (10%) responding with “3,” and one (3%) with “1.”

### Analysis of Free-Text survey questions

Free text questions were phrased to gather information in the participants’ own words about challenges or needs for integrating PPC into their courses (pretest survey), the *Essentials* domains on which they had focused, content used from the *Guide*, and how the *Learning Activity Template* was completed during the workshops (posttest survey).^1,20,21^

### Faculty challenges and needs

Of 40 participants who completed the pretest survey, four (10%) stated that they had no needs or challenges in incorporating PPC content into courses taught, including one certified PC nurse. Two participants (5%) identified a need for student access to clinical placements in palliative or hospice care. Twenty-five participants (63%) reported a need for faculty access to evidence-based resources and learning activities for use in live and asynchronous virtual settings. Within this 63% who reported a need, 4 participants had PC certification, 4 had completed an ELNEC training, and 2 had PC work experience without nationally recognized training. For those who were certified or had PC experience, this quote is representative of expressed needs: “I’m confident with the content. I’m not sure how to develop competency-based assessments.” Examples of other reported participant needs included lesson plans, case studies, simulations, guides for use in serious illness and advance care planning communication, competency-based assessment tools, guidance using the CARES competencies,^14,15^ or having an expert colleague available for consultation.

### Learning activity template categories

There was variation in PPC topical content focus when participants completed the *Learning Activity Template*.^21^ Seven of the 10 *Essentials* domains were used, incorporating 17 competencies and 28 subcompetencies for development of a learning activity.^1^ Twenty-four *Guide* resources were used in 28 unique learning activities^20^; Table 3 lists a selection of the resources used. Five examples of participants’ learning activities and the *Essentials* content with which they aligned are provided in Table 4.

**Table 3.** Learning Activity Template Themes from Posttest Questions

Essentials domains used in learning activities
Person-centered care (Domain 2)
Population health (Domain 3)
Scholarship for the nursing discipline (Domain 4)
Quality and safety (Domain 5)
Interprofessional partnerships (Domain 6)
Systems-based practice (Domain 7)
Informatics and health care technologies (Domain 8)
Professionalism (Domain 9)
Participant Learning Activity Topics
PC disparities and health equity
Pain management
Ethical care
Communication
Advance care planning
Shared decision-making
End of life
Lung cancer symptom management
Pediatric cultural and religious considerations
Technology in PC
Participant Learning Activity Titles
Exploring Internal and External Barriers to Equal Access to Hospice and Palliative Care
Practicing Empathy and Compassion
Role-Play Serious Illness Conversations with Patient/Nurse Roles
Using a PICOT Question and Literature Search for Evidence-Based Advanced Illness Symptom Management
Withdrawal of Life Sustaining Measures for a Client in Critical Care: A Pediatric Hospice Simulation

**Table 4.** Participant Learning Activity Template Examples

Participant number/example responses
*Essentials* domain selected
Participant 1: Domain 8 Informatics and Health care Technologies Participant 2: Domain 5 Quality and Safety Participant 3: Domain 2 Patient Centered Care Participant 4: Domain 3 Population Health Participant 5: Domain 6 Interprofessional Partnerships
*Essentials* competency selected Participant 1: 8.3 Use information and communication technologies and informatics processes to deliver safe nursing care to diverse populations in a variety of settings. Participant 2: 5.1 Apply quality improvement principles in care delivery. Participant 3: 2.2 Communicate effectively with individuals. Participant 4: 3.5 Demonstrate advocacy strategies. Participant 5: 6.3 Use knowledge of nursing and other professions to address health care needs
*Essentials* sub-competency selected Participant 1: 8.3a Demonstrate appropriate use of information and communication technologies. Participant 2: 5.1d Interpret benchmark and unit outcome data to inform individual and microsystem practice; 5.1f Identify strategies to improve outcomes of patient care in practice; 5.1 g Participate in the implementation of a practice change. Participant 3: 2.2a Demonstrate relationship-centered care; 2.2b Consider individual beliefs, values, and personalized information in communications; 2.2c Use a variety of communication modes appropriate for the context; 2.2d Demonstrate the ability to conduct sensitive or difficult conversations. Participant 4: 3.5i Demonstrate leadership skills to promote advocacy efforts that include principles of social justice, diversity, equity, and inclusion. Participant 5: 6.3a: Integrate the roles and responsibilities of health care professionals through interprofessional collaborative practice.
Learning activity topic area (e.g., pain management, heart failure) Participant 1: “Communication” Participant 2: “Communication, QI, DIKW, Technology, and any condition really.” Participant 3: “Communication” Participant 4: “Health Equity, Disparities, and Palliative Care” Participant 5: “Advanced care planning, cancer, serious illness”
Learning activity proposed name (e.g., Use of opioids for dyspnea relief in end-stage COPD): Participant 1: “Best Practices for Responding to Emotion-Respecting Communication with Diverse Populations” Participant 2: “Improving the Quality of Palliative Care Using Healthcare Information Technology” Participant 3: “Enhancing Communication Skills for Nurses in Caring for Patients with Serious Life Altering Illness” Participant 4: “Managing Implicit Bias and Its Effect on Health Care Disparities” Participant 5: “Palliative Care Module: Care for the Adult Patient with Advanced Cancer Through an Unfolding Case Study”
*Guidebook* linked resource used to support the learning activity: Participant 1: *Communication with Diverse Populations; Responding to Emotion-Respecting* Participant 2: *What is Palliative Care?* Participant 3: *Using the Serious Illness Conversation Guide; Communication with Diverse Populations* Participant 4: *Managing Implicit Bias and Its Effect on Health Care Disparities* Participant 5: *Debbie’s Palliative Care Story: An Update.* [Video].
Description of learning activity: Participant 1: “Students will review the PowerPoint ‘Communication with Diverse Populations’ and the ‘Responding to Emotion: Respecting’ document. They will be given a list of diverse patients and scenarios to choose from for the activity. The student will create a ‘Nursing Note’ that would be hypothetically documented in the patients EHR that would outline effective communication with the diverse patient (using criteria from the PowerPoint) …using the NURSE statements for articulating empathy.” Participant 2: “1) Identify healthcare informatics technologies that can be used to enhance evidence-based strategies to address a condition related to PC nursing. 2) Develop a project proposal to address the condition in PC nursing. 3) Diagram how the change will impact stake holder’s workflow and processes.” Participant 3: “Design a 2-part low fidelity simulation [using role-playing] for students that: 1) explores current error data from national websites related to quality outcomes. Students will use current national databases. 2) Engage in a role-playing simulation to practice having serious communication.” Participant 4: “1) View the 1-hour webinar and slide set in which Dr. Kimberly Curseen presents definitions of implicit bias and self-assessment, a framework for mitigating health care disparities using individuation and cultural humility, case scenarios, and suggestions for how clinicians can help resolve health inequities. 2) Participate in the discussion forum for this week and answer the question ‘What are some practical ways palliative care providers can begin to impact health disparities?’” Participant 5: “Students will be presented with an unfolding case study and complete a series of activities … in the care of the patient with a serious illness. Including holistic assessment, identifying and prioritization of problems, creation of a care plan, interprofessional involvement and matching roles and delegation of roles, patient communication, and evaluation of care. care of the dying and evaluation of own perspectives.”

## Discussion

This nursing faculty workshop intervention that reviewed hospice and palliative care definitions, basic elements of CBE learning activities, introduced faculty to the *Guide*, and taught participants how to use the *Learning Activity Template* to develop PPC CBE learning activities resulted in positive outcomes for participants.^20,21^ These included significant gains in knowledge about topical content essential to teaching PPC to nursing students. Additionally, participant attitudes/beliefs indicated significantly greater confidence in teaching end-of-life care and survey scores for the belief that PPC nursing education is an essential content area for prelicensure courses increased although not significantly.

When considering the wide range of workshop participant locations with 72% of U.S. states represented, it is apparent that nursing faculty across the country need guidance in locating evidence-based source materials and implementing content into baccalaureate courses. This was consistent with the self-identified needs reported by participants; only 7.5% reported specialty certification in palliative and hospice care, indicative that faculty feel the need to strengthen their teaching related to PPC. Of note, even those participants who reported confidence in teaching PPC in the pretest survey listed knowledge of CBE implementation (especially student assessment) as an area of need. Some of the needs reported in the preintervention survey had been met after participation, for example, all participants had identified a resource to use in learning activity development and several developed simulations and communication activities, which had been identified as priority needs. While we assessed a general faculty challenges question in the survey, there remains a need for a faculty PPC CBE needs assessment tool which can validly and reliably identify various types of needs. It was evident from pretest survey responses that most workshop participants did not have knowledge of or access to evidence-based resources to use in planning the integration and teaching of PPC content in their courses prior to the intervention, but there were significant gains in participant knowledge of PPC educational content after completing the workshop. Additional knowledge gains included significantly higher mean scores after the intervention workshop on understanding differences between hospice and palliative care; pain and dyspnea management; and empathetic communication. Mean scores increased but not significantly for pain and generalized symptom assessment; teaching culturally appropriate PPC; talking about dying; and discussing grief, bereavement, and loss. Faculty needs for teaching these topics would warrant further exploration. Importantly, by the end of the intervention workshop, 55% of participants had developed a complete PPC CBE learning activity, and 35% had developed most of a learning activity. All participants started the process of developing a PPC CBE learning activity.

The range of topics that participants selected for use in developing a CBE PPC learning activity is reflective of how nurse faculty comprehend the definition of palliative care—across age groups and serious illnesses, settings, and within a context of health inequities. It is also evident that as faculty participants work at home institutions to address the hospice/palliative/supportive care sphere, most *Essentials* domains will be represented.^1^ All domains will need to be addressed at each academic program as course revisions are made; however, faculty may determine which domains will address PPC.

Nursing’s holistic approach to care is reflected in the creative and diverse ways that study participants applied the resources and knowledge presented in the intervention workshop. Study limitations include a small, self-selected, homogenous sample and a survey instrument which lacked extensive validity testing. When interpreting our results, it must be considered that study participants self-selected both attendance at the workshop which offered incentives directly relevant to their teaching responsibilities and participation in the study, and thus with a population of faculty who were not responsible for integrating PPC in their programs, outcomes may have been different. Therefore, conclusions about generalizability of the study results may be limited. Furthermore, this study was based on a single time point intervention and evaluation, so conclusions about long-term impacts cannot be made. Although study results do not necessarily represent baccalaureate nursing faculty nationally, this study lays groundwork for future research with fully powered samples and provides nursing educators with important qualitative faculty input.

## Conclusion

Baccalaureate nursing faculty benefit from resources that support integration of the AACN *Essentials* hospice/palliative/supportive care sphere into programs and courses.^1^ The ELNEC undergraduate/new graduate modules fill a niche for online knowledge-based learning for students.^13^ However, faculty lack resources to use and guidance in developing CBE PPC learning activities.^12^ This intervention for faculty is an important step in meeting that need. Additional challenges for hospice/palliative/supportive care CBE integration in baccalaureate nursing education include a need for a variety of learning activities across each *Essentials* domain as well as assessment instruments for faculty needs and student competency.^1^ Research will be needed to evaluate efficacy of learning activities as well as validity and reliability of assessment instruments. This pilot study demonstrated how an intervention addressed one of these needs. Participants had positive outcomes that can influence coursework taught in the future across the United States. Importantly, and uniquely, the resources used in the intervention, as open-source digital items, remain freely available for any faculty to access and utilize.
